# Intrapulmonary penetration and pharmacokinetics of siderophore cephalosporin HRS-8427 injection in Chinese healthy subjects

**DOI:** 10.1128/aac.00702-25

**Published:** 2025-11-06

**Authors:** Yichen Cao, Yuanyuan Huang, Jinlian Xie, Qian Wu, Shuang Yang, Xiaoyan Yang, Jie Huang, Jinlong Liu, Jie Meng, Guoping Yang

**Affiliations:** 1 Clinical Pharmacology Center, The Third Xiangya Hospital of Central South University 504354, Changsha, Hunan, China; 2 Jiangsu Hengrui Pharmaceuticals Co, Ltd 425579 , Shanghai, China; 3 Xiangya School of Pharmaceutical Sciences, Central South University 506618 , Changsha, Hunan, China; 4 Department of Pulmonary and Critical Care Medicine, The Third Xiangya Hospital of Central South University 504354, Changsha, Hunan, China; 5 Hunan Key Laboratory of Organ Fibrosis, Changsha, Hunan, China; Providence Portland Medical Center, Portland, Oregon, USA

**Keywords:** HRS-8427, intrapulmonary pharmacokinetic, bronchoalveolar lavage, epithelial lining fluid

## Abstract

HRS-8427, a novel siderophore cephalosporin antibiotic, is under development for treating Gram-negative infections. This study evaluated the intrapulmonary penetration and pharmacokinetic (PK) profile of HRS-8427 injection following single and multiple dose administrations in healthy Chinese subjects. A single-center, randomized, open-label Phase 1 trial enrolled 38 subjects, with 35 completing the study. In the single-dose group, subjects received a 1,500 mg intravenous infusion of HRS-8427 over 2 h. In the multiple-dose group, subjects were administered 2,000 mg of HRS-8427 intravenously every 8 h for four consecutive doses, each infused over 2 h. Bronchoalveolar lavage (BAL) was conducted to collect epithelial lining fluid (ELF) and alveolar macrophage (AM) specimens. PK analysis demonstrated a maximum plasma concentration (*C*_max_) of 176 µg/mL and an area under the time-concentration curve from time zero to time infinity (AUC_0–∞_) of 942 h·μg/mL following the single 1,500 mg dose. After multiple 2,000 mg dosing, the *C*_max_ increased to 262 µg/mlL with a corresponding AUC_0–∞_ of 1,640 h·μg/mL. The ELF-to-plasma concentration ratio (*C*_ELF_/*C*_plasma_) exhibited a time-dependent increase, reaching 12.2% at 8 h post-final dose. Intracellular accumulation in AM remained minimal (<1%). Multiple-dose administration resulted in higher ELF exposure than single dosing. Safety assessments indicated an overall favorable tolerability profile, with most adverse events being mild in severity. Based on PK and microbiological data, a 2,000 mg regimen achieved concentrations exceeding the MIC₉₀ values of major Gram-negative pathogens, supporting further clinical development of HRS-8427 for respiratory and other Gram-negative bacterial infections.

**CLINICAL TRIALS:**

This study is registered with Chinese Clinical Trial Registry Platform as ChiCTR2300072350.

## INTRODUCTION

Gram-negative bacteria represent the predominant etiological agents of hospital-acquired bacterial infections (1). Clinically significant pathogens, including *Escherichia coli*, *Klebsiella pneumoniae*, *Pseudomonas aeruginosa*, and *Acinetobacter baumannii*, are implicated in polymicrobial infections spanning the urinary tract, respiratory system, and other critical organs (2, 3). Severe manifestations such as sepsis and septic shock contribute substantially to elevated in-hospital mortality rates. Current therapeutic strategies predominantly rely on antimicrobial agents such as fluoroquinolones, β-lactam/β-lactamase inhibitor combinations, carbapenems, and polymyxins. However, the escalating prevalence of multidrug-resistant (MDR) and extensively drug-resistant (XDR) strains has severely diminished the clinical efficacy of these regimens.

Gram-negative bacteria employ multifaceted resistance mechanisms, including the production of β-lactamases and aminoglycoside-modifying enzymes to enzymatically degrade or structurally modify antibiotics, thereby inactivating them (4, 5). Concurrently, these pathogens downregulate outer membrane porin expression to restrict antibiotic influx (6, 7). Their intrinsic resistance is further potentiated by a double-layered membrane architecture and lipopolysaccharide (LPS)-mediated endotoxin release, rendering most conventional antibiotics pharmacologically ineffective. Notably, the misuse and overuse of antibiotics have contributed to the global dissemination of MDR Gram-negative bacteria (8). Furthermore, disruption in prevention measures combined with the high rate of antibiotic use during the COVID-19 has substantially aggravated the rate of MDR pathogens (9). Epidemiological data show that the pooled prevalence of MDR *Klebsiella pneumoniae* in Southeast Asia has reached 55% (10), while a study in a tertiary hospital in Lebanon indicated that the prevalence of carbapenem-resistant *Escherichia coli* among hospitalized patients statistically significantly increased between 2013 and 2018 (11). Furthermore, the resistance rates of Gram-negative bacteria in ICU patients are much higher compared to non-ICU departments (12). MDR Gram-negative bacteria also exacerbate the nosocomial infection burden in oncological patients (13). This phenomenon has not only elevated mortality rates but also imposed substantial clinical and economic burdens (14). In the updated 2024 WHO list of priority pathogens, *Acinetobacter baumannii* and other Gram-negative bacteria resistant to last-line antibiotics are listed as critical priority pathogens (15). The evolution of resistance and the spread of Gram-negative bacteria within hospitals present significant treatment challenges, underscoring the urgent need for the development of novel antimicrobial agents to overcome existing therapeutic limitations.

Cefiderocol, a novel siderophore cephalosporin developed by Shionogi & Co., Ltd, targets Gram-negative bacteria, particularly carbapenem-resistant strains, through its unique siderophore-mediated active transport mechanism. Cefiderocol’s structural features are similar to those of ceftazidime and cefepime, and it exhibits high stability against various β-lactamases. Its catechol portion in the side chain chelates with iron and mimics natural siderophore molecules, allowing the drug to be actively transported across the bacterial outer membrane via specific iron transport proteins, achieving effective bactericidal concentrations in the bacterial periplasmic space (16, 17). Clinically, cefiderocol has demonstrated strong *in vitro* activity against Gram-negative bacteria, including *Pseudomonas aeruginosa*, *Klebsiella pneumoniae*, *Acinetobacter baumannii*, and *Stenotrophomonas maltophilia*. It has shown outstanding clinical cure rates and microbial clearance for complicated urinary tract infections and pulmonary infections (18). With the gradual introduction of cefiderocol into clinical practice, resistance among regional carbapenem-resistant *Enterobacteriaceae* and *Acinetobacter baumannii* has increased to 12.4% and 13.2%, but the overall resistance prevalence remains low (19). These findings suggest that although the limitations of existing therapeutics for MDR Gram-negative bacteria still remain, the development of novel siderophore cephalosporins may provide additional options for infection control.

HRS-8427 injection, a novel siderophore cephalosporin antibiotic independently developed by Jiangsu Hengrui Pharmaceutical Co., Ltd. It chelates extracellular iron ions and is actively transported into bacterial cells via iron transport proteins located in the bacterial outer membrane. Within the periplasmic space, HRS-8427 achieves elevated concentrations, binds to penicillin-binding proteins (PBPs), and inhibits cell wall biosynthesis, thereby exerting both bacteriostatic and bactericidal effects. Non-clinical studies have demonstrated that HRS-8427 exhibits potent antibacterial activity against aerobic Gram-negative pathogens, including *Enterobacteriaceae*, *Pseudomonas aeruginosa*, and *Acinetobacter baumannii*. In a murine urinary tract infection model, HRS-8427 significantly reduced bacterial burdens in both the bladder and kidneys. Furthermore, preclinical findings indicated that the bactericidal activity of HRS-8427 increased with dosing frequency, suggesting a time-dependent killing profile. (All data referenced are unpublished).

To date, HRS-8427 has completed a Phase I study evaluating its safety, tolerability, and pharmacokinetic (PK) (Study HRS-8427-101; Chictr.org.cn identifier ChiCTR2200062570), a PK study in subjects with renal impairment (ClinicalTrials.gov identifier NCT07070375), and a dose-escalation study in patients with complicated urinary tract infections (ClinicalTrials.gov identifier NCT06144060). A Phase III clinical trial in patients with complicated urinary tract infections is currently ongoing (ClinicalTrials.gov identifier NCT06569056). All data are currently unpublished.

The primary aim of this study is to evaluate the intrapulmonary penetration and PK profile of HRS-8427 injection following both single and multiple-dose administrations in healthy Chinese subjects.

## MATERIALS AND METHODS

### Study design

This was a single-center, randomized, open-label Phase I clinical trial designed to evaluate the intrapulmonary penetration and PK profile of HRS-8427 following single and multiple intravenous administrations in healthy adult subjects. The study protocol was approved by the Ethics Committee of Xiangya Third Hospital, Central South University, and written informed consent was obtained from all participants. The study consisted of two parts. The first part was a single-dose study in which 20 subjects were randomly assigned to Groups A–D (1:1:1:1) to receive a single 1,500 mg intravenous infusion of HRS-8427 (infusion duration: 120  ±  5 min). Bronchoalveolar lavage (BAL) was conducted at 2, 3, 5, and 8 h post-dose to collect epithelial lining fluid (ELF) samples. The second part was a multiple-dose study in which 15 subjects were allocated to Groups E, F, and G (5 subjects per group), receiving 2,000 mg of HRS-8427 intravenously every 8 h for a total of four doses. BAL was performed at 2, 4, and 8 h after the final dose to collect BAL fluid. The selection of BAL time points in the multi-dose group was based on single-dose data that indicated comparable ELF concentrations at 3 and 5 h. Combining these time points at 4 h could effectively characterize the pulmonary PK profile while reducing the number of BAL procedures required and subject burden. The initiation of the study for Groups F and G was determined jointly by the investigator and the sponsor, and a parallel group design was employed.

The dose selection for the single- and multiple-dose groups was based on prior pharmacokinetics/pharmacodynamics (PK/PD) modeling and emerging PK data. The 1,500 mg single-dose level was chosen based on unpublished preclinical PK/PD simulations, which suggested that this dose could achieve clinically relevant exposures with a thrice-daily dosing regimen. However, preliminary data from the single-dose study revealed that the intrapulmonary penetration of HRS-8427 was lower than anticipated, with ELF concentrations unlikely to reach effective levels for treating pneumonia. Based on these findings and the favorable safety profile in previous multiple ascending dose (MAD) studies (unpublished data), a 2,000 mg q8h dosing regimen was selected for further investigation in the multiple-dose group. This dose escalation aimed to characterize the pulmonary exposure and PK profile of HRS-8427 at a higher dose level potentially effective for respiratory infections.

Eligible subjects were admitted to the research center 1 day prior to the initiation of dosing (Day −1) for screening and baseline assessments. Subjects in the single-dose group underwent PK blood sampling and safety evaluations at predefined time points before and after dosing. They were discharged on Day 2 and returned on Day 4 for a follow-up safety assessment. Subjects in the multiple-dose group completed all scheduled procedures and safety assessments before being discharged on Day 3. A telephone follow-up was conducted on Day 8  ±  1.

### Study population

Subjects were healthy adults aged 18–45 years with a body mass index (BMI) between 19.0 and 26.0 kg/m². All participants were required to fully understand the study and voluntarily consent to participate. Eligible male subjects weighed ≥50.0 kg, and female subjects weighed ≥45.0 kg, with no plans for pregnancy within 3 months following study completion. Exclusion criteria included: any diseases or surgical history that could interfere with PK evaluations or increase the risk of procedures; use of any prescription, over-the-counter drugs, herbal medicine, or vitamins within the specified time before screening; smoking, excessive alcohol consumption, or excessive intake of caffeine-containing beverages; participation in other clinical trials, vaccination, or blood donation within the specified period before screening; history of asthma or food/drug allergies; drug abuse or illicit drug use; inability to tolerate or undergo BAL; inability to follow the controlled diet; female subjects who were pregnant or breastfeeding; and subjects with any clinically significant laboratory abnormalities.

### Blood sample collection

In the single-dose group, venous blood samples were collected within 1 h prior to dosing and at 1, 2 (immediately after infusion), 2.5, 3, 4, 5, 6, 8, 10, 12, and 24 h post-dose. In the multiple-dose group, blood samples were collected at the same time points as in the single-dose group, with additional sampling at 5 min before the final dose and at 1, 2 (immediately after infusion), 2.5, 3, 4, 5, 6, 8, 10, 12, and 24 h after the final dose. At each time point, 3 mL of venous blood was collected for PK analysis and another 3 mL for urea concentration measurement, using tubes containing anticoagulants. The time points for urea sampling were aligned with those for BAL collection: 2, 3, 5, and 8 h after administration in the single-dose group, and 2, 4, and 8 h in the multiple-dose group. Following collection, blood samples were centrifuged within 30 min for 10 min to obtain plasma. The plasma was transferred into cryovials and stored at −60°C to −90°C within 1 h after centrifugation. Backup samples were also preserved for subsequent analysis.

### BAL

Each subject underwent a single BAL procedure at a designated time point during the study. Single-dose group subjects underwent BAL at 2, 3, 5, and 8 h post-dose, while multiple-dose group subjects underwent BAL at 2, 4, and 8 h after the final dose. BAL was performed three times at each time point. For each lavage, 50 mL of sterile normal saline was instilled into the subsegmental bronchus of the right middle lobe and immediately aspirated. The second to third lavage fluids were collected, pooled, and subjected to subsequent analyses. All BAL fluids were immediately placed on ice and processed within 2 h of collection.

BAL samples were centrifuged at 4°C, 400 g for 5 min. Subsequently, 4 mL of the supernatant was aliquoted into cryovials for PK analysis of HRS-8427 and pharmacodynamic analysis of urea concentration in ELF. The remaining cells were preserved for further evaluation. ELF samples were stored at −60°C to −90°C until transport.

Following separation of the supernatant, 5 mL of 0.9% saline solution was added to the BAL collection tube to resuspend the cell pellet. The resulting suspension was then transferred into a 15 mL centrifuge tube and centrifuged at 400 g for 5 min at 4°C. After centrifugation, the supernatant was carefully removed, and the remaining cell pellet, designated as the alveolar macrophage (AM) sample, was stored at −60°C to −90°C until transportation.

### Determination of HRS-8427 concentrations in plasma, BAL fluid, and AM

Plasma concentrations of HRS-8427 were determined using a validated Liquid Chromatography-Tandem Mass Spectrometry (LC-MS/MS) assay at an independent analytical facility. The calibration curve exhibited linearity over the range of 1.00–600 μg/mL, with quality control (QC) samples prepared at high (450 µg/mL), medium (200 µg/mL), low-medium (25.0 µg/mL), and low (2.50 µg/mL) concentrations. Intra- and inter-run precision were <9.5% across all QC levels, while precision at the lower limit of quantification (LLOQ, 1.00 µg/mL) was <9.3%. Matrix effects, evaluated via internal standard (cefiderocol)-normalized matrix factors, ranged from 105.9% to 109.2% with ≤1.4% coefficients of variation. Extraction recoveries were 98.4%–102.0% for HRS-8427 and 101.1% for the internal standard.

A validated LC-MS/MS method was employed for BAL fluid analysis, demonstrating linearity from 1.00 to 200 ng/mL. QC samples included high (150 ng/mL), medium (80.0 ng/mL), low-medium (30.0 ng/mL), and low (3.00 ng/mL) concentrations. Intra- and inter-run precision were <11.2% across all QC levels, while precision at the LLOQ was <9.1%. In the assessment of matrix effects, the mean deviation of the measured values from theoretical values for low-concentration quality control (LQC) samples was −0.3%, with a corresponding precision of 4.6%. For high-concentration quality control (HQC) samples, the mean deviation was −0.7%, with a precision of 4.3%. The extraction recovery of HRS-8427 ranged from 87.6% to 96.3%, while that of the internal standard 73500 was 101.7%.

AM samples were analyzed using a validated LC-MS/MS assay with a linear range of 1.00–200 ng/mL. QC concentrations included high (150 ng/mL), medium (80.0 ng/mL), and low (3.00 ng/mL) levels. Intra- and inter-run precision were <5.9% across all levels and <5.4% at LLOQ (1.00 ng/mL).

### Determination of urea concentration

Plasma urea concentrations were quantified using a validated LC-MS/MS method. The calibration curve exhibited good linearity across the range of 30.0 to 900 µg/mL. QC samples at high (675 µg/mL), medium (450 µg/mL), low-medium (150 µg/mL), and low (90.0 µg/mL) concentrations demonstrated intra- and inter-run precision of less than 7.8%. At the LLOQ (30.0 µg/mL), the intra- and inter-run precision was below 7.7%. For matrix effect evaluation, the mean relative bias was 3.5% at the LQC level and 0.3% at the HQC level, with corresponding precision values of 6.3% and 4.7%. The extraction recovery of urea ranged from 92.8% to 95.6%, while that of the internal standard ([^13^C;^15^N₂]-urea) was 94.9%.

Urea concentrations in BAL fluid were measured using a validated LC-MS/MS method. The calibration curve showed good linearity in the range of 0.500 to 20.0 µg/mL. QC samples were prepared at concentrations of 15.0, 6.00, 3.00, and 1.50 µg/mL, representing high, medium, low-medium, and low levels, respectively. The intra- and inter-run precision for all QC levels was less than 13.0%, and at the LLOQ (0.500 µg/mL), it was below 15.0%. In matrix effect assessments, the mean deviation between measured and theoretical values at the low-medium QC level was −1.2% with a precision of 2.8%, and at the HQC level, the mean deviation was −2.7% with a precision of 2.7%. The extraction recovery of urea ranged from 98.6% to 106.1%, and the recovery of the internal standard ([^13^C;^15^N₂]-urea) was 99.2%.

### Calculation of HRS-8427 concentrations in ELF and AM

The apparent volume of ELF in BAL fluid was estimated using the urea nitrogen dilution method (20, 21). The concentration of HRS-8427 in ELF was calculated using the formula: *C*_ELF_ = *C*_BALF_ × (urea_plasma_ /urea_BALF_), where *C*_BALF_ is the measured concentration of HRS-8427 in BAL fluid, and urea_plasma_ and urea_BALF_ represent the measured concentrations of urea in plasma and BAL fluid, respectively. Absolute and differential cell counts were obtained from BAL fluid. The concentration of HRS-8427 in AM was calculated as: *C*_AM_ = *C*_suspension_/ _VAM_, where *C*_suspension_ is the concentration of HRS-8427 in the cell suspension and *V*_AM_ is the estimated total volume of alveolar cells. The calculation of *V*_AM_ was based on a mean macrophage volume of 2.42 µL per 10⁶ cells (22, 23).

### Statistical analysis

The PK statistical analysis was performed using the CPhaMAS platform (24) (V1.0, https://www.cphamas.cn/). PK parameters for HRS-8427 in plasma, ELF, and AM were calculated, including time to maximum concentration (*T*_max_), maximum plasma concentration (*C*_max_), area under the time-concentration curve from time zero to time *t* (AUC_0*–t*_), area under the time-concentration curve from time zero to time infinity (AUC_0–∞_), elimination half-life (*T*_1/2_), terminal elimination clearance (CL_z_), and volume of distribution during the terminal phase (*V*_z_). ELF and AM PK parameters were calculated as ratios to plasma concentrations. All statistical analyses were performed using SAS (Version 9.4) for PK concentration data, *c*-*t* curve plotting, and safety assessments.

### Safety assessments

Safety was assessed through a comprehensive evaluation that included physical examinations, monitoring of vital signs, 12-lead electrocardiogram (ECG), and a series of laboratory tests, including blood routine, blood biochemistry, urine routine, coagulation profile, and pregnancy tests (for female subjects). Adverse events (AEs) and serious adverse events (SAEs) were closely monitored throughout the study.

## RESULTS

### Population

**Fig 1:**
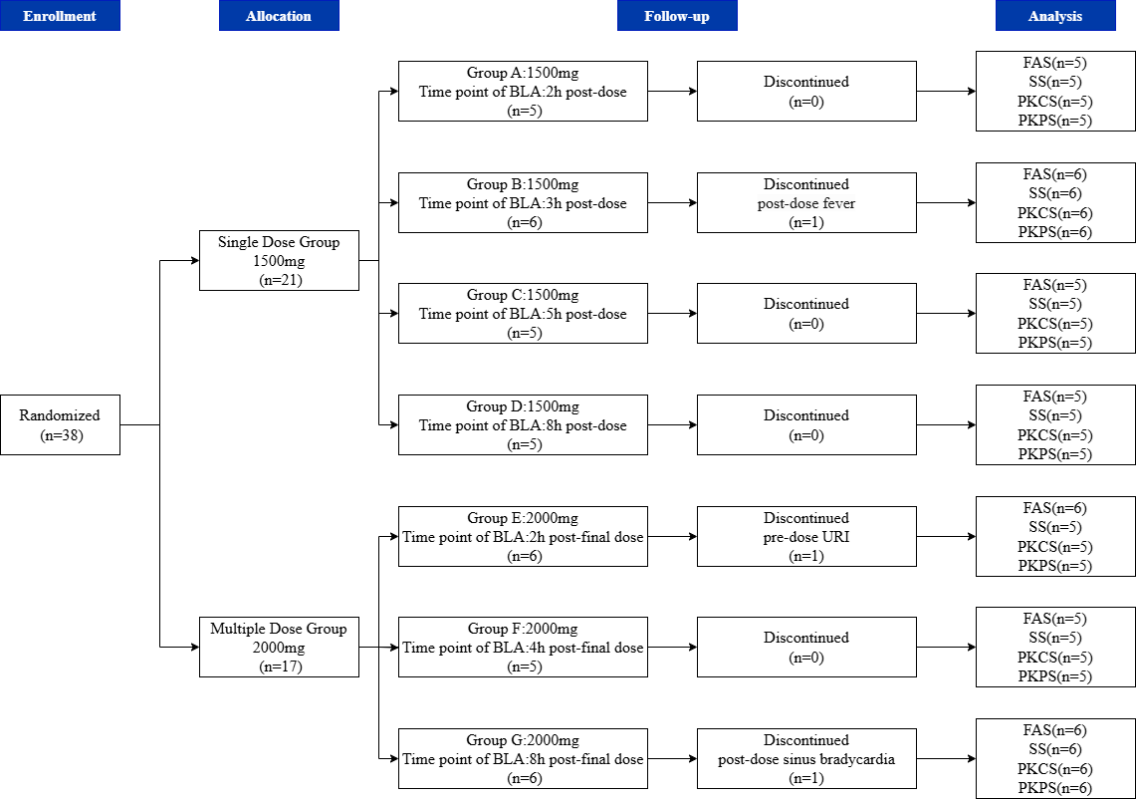
Disposition of all subjects. URI, upper respiratory tract infection; FAS, full analysis set; SS, safety set; PKCS, pharmacokinetics concentration set; PKPS, pharmacokinetics parameter set.

A total of 38 subjects were enrolled in this study, with 3 subjects withdrawing prior to study completion. One subject withdrew before dosing and was included in the Full Analysis Set (FAS). Two subjects withdrew after dosing and did not undergo BAL. Therefore, they were excluded from the BAL PK analysis. Fig. 1 illustrates the disposition of all subjects.

Baseline demographic characteristics of the 38 subjects included in the FAS are summarized in Table 1. Among them, 27 were male (71.1%) and 11 were female (28.9%). The mean age of the subjects was 26.7 years, with a mean body mass index (BMI) of 22.46 ± 2.02 kg/m². In the single-dose group (1,500 mg), the mean age was 25.4 years and the mean BMI was 22.81 ± 2.02 kg/m². In the multiple-dose group (2,000 mg), the mean age was 28.4 years and the mean BMI was 21.99 ± 1.98 kg/m². The demographic characteristics between the two study groups were generally comparable.

**TABLE 1:** Summary of demographic and baseline characteristics grouped by single and multiple administration^*a*^

	SAD 1,500 mg (*N* = 21)	MAD 2,000 mg (*N* = 17)	Total (*N* = 38)
Age (years)			
*n*	21	17	38
Mean (SD)	25.4 (4.31)	28.4 (5.24)	26.7 (4.91)
Median	25.0	28.0	26.0
Min, Max	21, 36	21, 41	21, 41
Sex, *n* (%)			
Male	11 (52.4)	16 (94.1)	27 (71.1)
Female	10 (47.6)	1 (5.9)	11 (28.9)
Ethnicity, *n* (%)			
Han nationality	21 (100.0)	14 (82.4)	35 (92.1)
Other	0 (0.0)	3 (17.6)	3 (7.9)
Height (cm)			
*n*	21	16	37
Mean (SD)	164.19 (8.34)	167.94 (6.15)	165.81 (7.61)
Median	167.50	167.25	167.50
Min, Max	150.5, 175.5	159.5, 177.5	150.5, 177.5
Weight (kg)			
*n*	21	16	37
Mean (SD)	61.59 (7.67)	61.99 (5.94)	61.76 (6.88)
Median	61.80	61.70	61.80
Min, Max	46.0, 73.7	52.6, 70.9	46.0, 73.7
BMI (kg/m^2^)			
*n*	21	16	37
Mean (SD)	22.81 (2.02)	21.99 (1.98)	22.46 (2.02)
Median	22.70	21.40	22.20
Min, Max	20.0, 25.7	19.5, 25.4	19.5, 25.7

^*a*^ SAD, single ascending dose; MAD, multiple ascending dose; SD, standard deviation; BMI, body mass index.

### PK

**Fig 2:**
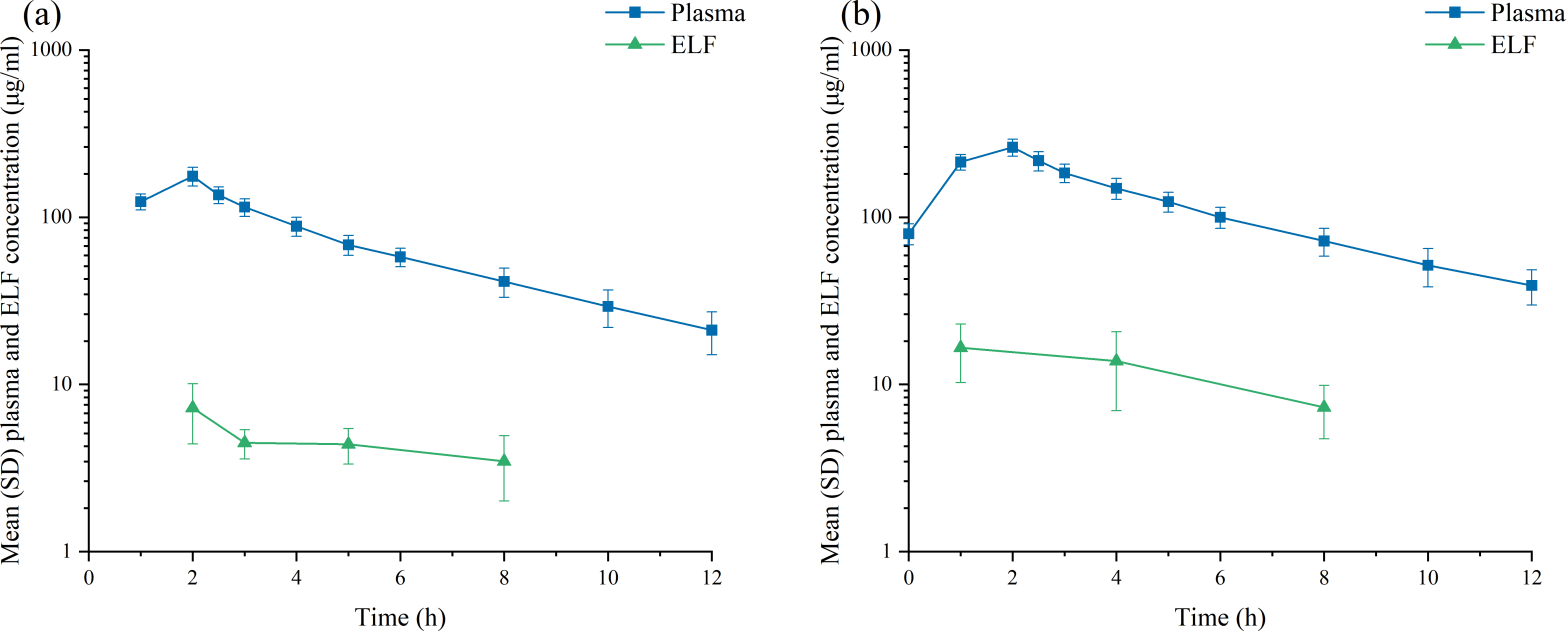
(**a**) Mean (SD) concentration-time profiles of HRS-8427 in plasma and ELF following single intravenous infusion of 1,500 mg HRS-8427. (**b**) Mean (SD) concentration-time profiles of HRS-8427 in plasma and ELF following multiple intravenous infusion of 2,000 mg HRS-8427. *SD, standard deviation; ELF, epithelial lining fluid.

The plasma PK data and ELF concentration-time curve following a single 2 h intravenous infusion of 1,500 mg HRS-8427 are presented in Fig. 2. HRS-8427 in both plasma and ELF reached peak concentrations at 2 h after infusion, with a mean *C*_max_ of 176 µg/mL and an average AUC_0–∞_ of 942 h·μg/mL. After 2–8 h of administration, the concentration of HRS-8427 in ELF ranged from 3.47 to 7.28 µg/mL and gradually decreased with prolonged administration time.

In subjects who received multiple 2 h intravenous infusions of 2,000 mg HRS-8427, peak concentrations in both plasma and ELF were also observed at 2 h post-infusion. The mean steady-state *C*_max_ (*C*_max,ss_) was 262 µg/mL, and the mean steady-state AUC_0–∞,ss_ was 1,640 h·μg/mL. The area ratio of ELF to plasma exposure (AUC_ELF_/AUC_plasma_) over the 0–8 h time concentration curve was 8.86%. At 2, 4, and 8 h following the final dose, ELF concentrations of HRS-8427 were 16.6 µg/mL, 13.8 µg/mL, and 7.3 µg/mL, respectively (Fig. 2). Other PK parameters are shown in Table 2.

**TABLE 2:** HRS-8427 pharmacokinetic parameters following single-dose and multiple-dose group^*a*^

Parameter	Plasma (mean ± SD)		ELF (mean)	
	SAD *n* = 21	MAD *n* = 16	SAD *n* = 21	MAD *n* = 16
*C*_max_(μg/mL)	176 ± 22.4	262 ± 30.8	7.24	16.58
*T*_max_(h)	1.99 ± 0.0410	1.96 ± 0.0319	2	2
AUC_0–t_(h·μg/mL)	917 ± 134	1,610 ± 232	33.58	87.76
AUC_0–∞_(h·μg/mL)	942 ± 140	1,640 ± 244	99.65	NA
*T*_1/2_(h)	4.06 ± 0.701	4.27 ± 0.404	13.18	NA

^*a*^ SAD, single ascending dose; MAD, multiple ascending dose; SD, standard deviation; *C*_max_, maximum plasma concentration; *T*_max_, time to maximum concentration; AUC_0–t_, area under the time-concentration curve from time zero to time *t*; AUC_0–∞_, area under the time-concentration curve—from time zero to time infinity; *T*_1/2_, elimination half-life; NA, not applicable.

AM PK data are presented in Table 3. Following a single 1,500 mg dose of HRS-8427, AM concentrations ranged from 0.246 to 0.320 µg/mL between 2 and 8 h post-dose. The *C*_AM_/*C*_plasma_ ratios at 2, 3, 5, and 8 h were 0.186%, 0.222%, 0.39%, and 0.693%, respectively. After four consecutive doses of 2,000 mg, AM concentrations ranged from 0.288 to 1.19 µg/mL. At 2, 4, and 8 h post-dose, the *C*_AM_/*C*_plasma_ ratios were 0.291%, 0.835%, and 0.428%, respectively. These findings suggest that HRS-8427 exhibits limited uptake by AM.

**TABLE 3:** Concentration of HRS-8427 in AM (μg/mL)^*a*^

Descriptive statistics	SAD 1,500 mg	SAD 1,500 mg	SAD 1,500 mg	SAD 1,500 mg	MAD 2,000 mg	MAD 2,000 mg	MAD 2,000 mg
	2 h	3 h	5 h	8 h	2 h	4 h	8 h
*n*	5	5	5	5	5	5	5
Mean	0.320	0.246	0.264	0.276	0.754	1.19	0.288
SD	0.384	0.331	0.307	0.326	0.348	1.28	0.348
CV%	119.9	134.7	116.2	118.0	46.2	107.6	120.7
Median	0.140	0.110	0.0700	0.0600	0.870	0.670	0.0700
Min	0.0300	0.0200	0.0200	0.0200	0.390	0.510	0.0100
Max	0.970	0.810	0.710	0.740	1.20	3.48	0.760
GeoMean	0.171	0.0992	0.122	0.122	0.684	0.868	0.104
GeoCV%	215.5	360.3	289.1	313.0	54.1	93.1	520.6

^*a*^ SAD, single ascending dose; MAD, multiple ascending dose; SD, standard deviation; CV, coefficient of variation.

### Safety

In this study, a total of 37 subjects received HRS-8427. Among them, 29 subjects (78.4%) experienced 66 treatment-emergent adverse events (TEAEs), and 5 subjects (13.5%) experienced 9 treatment-related adverse events (TRAEs). In the single-dose group of 21 subjects, 16 subjects (76.2%) experienced 35 TEAEs, and 3 subjects (14.4%) experienced 4 TRAEs. TEAEs reported in more than one subject included elevated blood pressure diastolic increased (14.3%, 3/21), blood pressure diastolic increased (9.5%, 2/21), bacterial test positive (9.5%, 2/21), oropharyngeal discomfort (47.6%, 10/21), cough (23.8%, 5/21), and expectoration (9.5%, 2/21). Among the 16 subjects in the multiple-dose group, 13 subjects (81.3%) experienced 31 TEAEs, and 2 subjects (12.5%) experienced 5 TRAEs. TEAEs that occurred in more than one subject included blood pressure diastolic increased (25.0%, 4/16), blood glucose decreased (12.5%, 2/16), oropharyngeal discomfort (25.0%, 4/16), cough (12.5%, 2/16), and sinus bradycardia (12.5%, 2/16). Cough, expectoration, and oropharyngeal discomfort were mainly attributed to discomfort following BAL. Bacterial test positive were mostly related to urine bacterial culture positivity, which may have resulted from sample contamination. Detailed information is presented in Table 4.

**TABLE 4:** TEAEs are classified and summarized by system organs and preferred terms

	Single-dose group				Multiple-dose group		
System organ classification (SOC）	1,500 mg—2 h (*N* = 5)	1,500 mg—3 h (*N* = 6)	1,500 mg—5 h (*N* = 5)	1,500 mg—8 h (*N* = 5)	2,000 mg—2 h (*N* = 5)	2,000 mg—4 h (*N* = 5)	2,000 mg—8 h (*N* = 6)
Preferred term (PT）	*n* (%)	*n* (%)	*n* (%)	*n* (%)	*n* (%)	*n* (%)	*n* (%)
Number of subjects with at least one adverse event	3 (60.0)	5 (83.3)	4 (80.0)	4 (80.0)	5 (100.0)	2 (40.0)	6 (100.0)
General disorders and administration site conditions	0 (0.0)	1 (16.7)	0 (0.0)	0 (0.0)	1 (20.0)	0 (0.0)	1 (16.7)
Chest discomfort	0 (0.0)	0 (0.0)	0 (0.0)	0 (0.0)	0 (0.0)	0 (0.0)	1 (16.7)
Fever	0 (0.0)	1 (16.7)	0 (0.0)	0 (0.0)	0 (0.0)	0 (0.0)	0 (0.0)
Administration site pain	0 (0.0)	0 (0.0)	0 (0.0)	0 (0.0)	1 (20.0)	0 (0.0)	0 (0.0)
Investigations	0 (0.0)	2 (33.3)	4 (80.0)	3 (60.0)	2 (40.0)	1 (20.0)	6 (100.0)
Blood pressure diastolic increased	0 (0.0)	0 (0.0)	2 (40.0)	1 (20.0)	0 (0.0)	1 (20.0)	3 (50.0)
Blood glucose decreased	0 (0.0)	0 (0.0)	0 (0.0)	0 (0.0)	0 (0.0)	0 (0.0)	2 (33.3)
Heart rate increased	0 (0.0)	1 (16.7)	0 (0.0)	0 (0.0)	0 (0.0)	0 (0.0)	1 (16.7)
White blood cell count increased	0 (0.0)	0 (0.0)	0 (0.0)	0 (0.0)	0 (0.0)	0 (0.0)	1 (16.7)
Blood uric acid increased	0 (0.0)	0 (0.0)	2 (40.0)	0 (0.0)	0 (0.0)	0 (0.0)	1 (16.7)
Urinary sediment present	0 (0.0)	0 (0.0)	0 (0.0)	1 (20.0)	0 (0.0)	0 (0.0)	0 (0.0)
White blood cells urine positive	0 (0.0)	0 (0.0)	0 (0.0)	1 (20.0)	0 (0.0)	0 (0.0)	0 (0.0)
Red blood cells urine positive	0 (0.0)	1 (16.7)	0 (0.0)	0 (0.0)	0 (0.0)	0 (0.0)	0 (0.0)
Electrocardiogram T wave abnormal	0 (0.0)	0 (0.0)	1 (20.0)	0 (0.0)	0 (0.0)	0 (0.0)	0 (0.0)
Bacterial test positive	0 (0.0)	1 (16.7)	0 (0.0)	1 (20.0)	0 (0.0)	0 (0.0)	0 (0.0)
Bile acids increased	0 (0.0)	0 (0.0)	0 (0.0)	0 (0.0)	1 (20.0)	0 (0.0)	0 (0.0)
Blood pressure decreased	0 (0.0)	0 (0.0)	0 (0.0)	0 (0.0)	1 (20.0)	0 (0.0)	0 (0.0)
Blood triglycerides increased	0 (0.0)	0 (0.0)	0 (0.0)	1 (20.0)	0 (0.0)	0 (0.0)	0 (0.0)
Blood potassium decreased	0 (0.0)	1 (16.7)	0 (0.0)	0 (0.0)	0 (0.0)	0 (0.0)	0 (0.0)
Nervous system disorders	0 (0.0)	0 (0.0)	0 (0.0)	1 (20.0)	0 (0.0)	0 (0.0)	1 (16.7)
Dizziness	0 (0.0)	0 (0.0)	0 (0.0)	1 (20.0)	0 (0.0)	0 (0.0)	1 (16.7)
Respiratory, thoracic, and mediastinal disorders	3 (60.0)	3 (50.0)	2 (40.0)	2 (40.0)	3 (60.0)	0 (0.0)	2 (33.3)
Oropharyngeal discomfort	3 (60.0)	3 (50.0)	2 (40.0)	2 (40.0)	2 (40.0)	0 (0.0)	2 (33.3)
Hiccups	0 (0.0)	0 (0.0)	0 (0.0)	0 (0.0)	0 (0.0)	0 (0.0)	1 (16.7)
Tachypnoea	0 (0.0)	0 (0.0)	0 (0.0)	0 (0.0)	0 (0.0)	0 (0.0)	1 (16.7)
Cough	1 (20.0)	2 (33.3)	2 (40.0)	0 (0.0)	2 (40.0)	0 (0.0)	0 (0.0)
Expectoration	0 (0.0)	1 (16.7)	1 (20.0)	0 (0.0)	0 (0.0)	0 (0.0)	0 (0.0)
Sneezing	0 (0.0)	0 (0.0)	0 (0.0)	0 (0.0)	1 (20.0)	0 (0.0)	0 (0.0)
Nasal obstruction	0 (0.0)	0 (0.0)	0 (0.0)	0 (0.0)	1 (20.0)	0 (0.0)	0 (0.0)
Cardiac disorders	0 (0.0)	1 (16.7)	0 (0.0)	0 (0.0)	1 (20.0)	0 (0.0)	2 (33.3)
Palpitations	0 (0.0)	0 (0.0)	0 (0.0)	0 (0.0)	0 (0.0)	0 (0.0)	1 (16.7)
Sinus bradycardia	0 (0.0)	1 (16.7)	0 (0.0)	0 (0.0)	1 (20.0)	0 (0.0)	1 (16.7)
Infections and infestations	0 (0.0)	0 (0.0)	0 (0.0)	0 (0.0)	0 (0.0)	0 (0.0)	1 (16.7)
Upper respiratory tract infection	0 (0.0)	0 (0.0)	0 (0.0)	0 (0.0)	0 (0.0)	0 (0.0)	1 (16.7)
Psychiatric disorders	0 (0.0)	0 (0.0)	0 (0.0)	0 (0.0)	1 (20.0)	0 (0.0)	0 (0.0)
Fear of injection	0 (0.0)	0 (0.0)	0 (0.0)	0 (0.0)	1 (20.0)	0 (0.0)	0 (0.0)
Gastrointestinal disorders	0 (0.0)	0 (0.0)	0 (0.0)	0 (0.0)	0 (0.0)	1 (20.0)	1 (16.7)
Nausea	0 (0.0)	0 (0.0)	0 (0.0)	0 (0.0)	0 (0.0)	0 (0.0)	1 (16.7)
Glossodynia	0 (0.0)	0 (0.0)	0 (0.0)	0 (0.0)	0 (0.0)	1 (20.0)	0 (0.0)
Blood and lymphatic system disorders	0 (0.0)	1 (16.7)	0 (0.0)	0 (0.0)	0 (0.0)	0 (0.0)	0 (0.0)
Lymphadenitis	0 (0.0)	1 (16.7)	0 (0.0)	0 (0.0)	0 (0.0)	0 (0.0)	0 (0.0)

Of the 66 TEAEs, 63 were mild in severity, and 3 were moderate, including lymphadenitis, blood pressure decreased, and upper respiratory tract infection. Lymphadenitis was determined to be unrelated to the study drug. Two subjects withdrew from the study prematurely due to fever and sinus bradycardia. Both events were classified as mild in severity. The sinus bradycardia was assessed and determined to be unrelated to the study drug, and fever was considered a TRAE.

## DISCUSSION

This study is the first to assess the intrapulmonary penetration and PK characteristics of HRS-8427 following intravenous administration in healthy adults. In the previous HRS-8427-101 study, we investigated multiple 2,000 mg intravenous infusions over 1 h. In the 101 study, the mean *C*_max,ss_ of HRS-8427 was 298 µg/mL, which represented a 13.7% increase compared to the current study. The mean AUC_0–∞,ss_ of HRS-8427 was 1,320 h·μg/mL, which was 19.5% lower than in this study (unpublished data). These results suggest that extending the infusion time may optimize the exposure characteristics of HRS-8427. Additionally, in the single-dose study of the present research, following a 2 h intravenous infusion of 1,500 mg of HRS-8427, with a mean *T*_1/2_ of 4.06 h and a mean AUC_0–∞_ of 942 h·μg/mL. In comparison, following a single 1 h intravenous infusion of 1,500 mg of cefiderocol, the mean *T*_1/2_ was 2.26 h, and the AUC_0–∞_ was 168.1 h·μg/mL. In the multiple-dose group, following continuous infusion of 2,000 mg HRS-8427 every 8 h over a 2 h period, the mean plasma *T*_1/2_ was 4.27 h, and the mean AUC_0–∞_ was 1,640 h·μg/mL. At the same dose, multiple 1 h infusions of cefiderocol every 8 h resulted in a mean plasma *T*_1/2_ of 2.40 h and a mean AUC_0–∞_ of 338.5 h·μg/mL (25). These findings indicate that, at the same dose, the *T*_1/2_ of HRS-8427 is approximately 1.8 times longer than that of cefiderocol, and the mean AUC_0–∞_ is approximately 4.8–5.6 times higher, suggesting that the clinically effective dose of HRS-8427 may be lower than that of cefiderocol. Furthermore, the prolonged *T*_1/2_ may reduce dosing frequency, thereby improving patient compliance and potentially expanding its applicability in patients with renal insufficiency.

The mean *C*_max_ of ELF in the 1,500 mg single-dose group was 7.24 µg/mL, and the mean AUC_0–∞_ was 99.65 h·μg/mL. In the 2,000 mg multiple-dose group, the mean *C*_max_ of ELF was 16.58 µg/mL. In comparison, following a single 2,000 mg infusion of cefiderocol, the mean ELF *C*_max_ was 13.8 µg/mL, and the mean AUC_0–∞_ was 33.12 h·μg/mL (26). Compared with cefiderocol, the ELF concentrations achieved following multiple-dose administration of HRS-8427 were sufficient to exceed the MIC₉₀ values of major Gram-negative pathogens, including *Klebsiella pneumoniae* (MIC₉₀ = 8 µg/mL) (27), *Pseudomonas aeruginosa* (MIC₉₀ = 0.5 µg/mL), and *Acinetobacter baumannii* (MIC₉₀ = 1 µg/mL) (28). In contrast, a single 1,500 mg dose resulted in ELF concentrations that did not fully cover the MIC₉₀ range of these organisms (MIC₉₀ ≤ 8 µg/mL). Additionally, in the single-dose study, HRS-8427 demonstrated low pulmonary permeability, and the rate of concentration change in ELF was inconsistent with that in plasma, with the decline in ELF concentration being slower than its plasma elimination. Following multiple administrations, both the pulmonary permeability and ELF concentrations of HRS-8427 increased. The ratios of ELF concentration to plasma concentration (*C*_ELF_/*C*_plasma_) at corresponding time points were 6.29%, 8.90%, and 12.2%, respectively. Based on these findings, a 2,000 mg dose appears to represent a potentially effective regimen for the treatment of pulmonary infections with HRS-8427. However, this requires confirmation in subsequent clinical trials.

The PK results of HRS-8427 in AM indicated that, following both single and multiple dosing, the concentration of HRS-8427 in AM was much lower than in ELF, suggesting limited active uptake by AM. After a single 2 h intravenous infusion of 1,500 mg HRS-8427, the *C*_max_ in AM was 0.86 µg/mL, and the AUC_0–8_ was 3.54 h·μg/mL. Following four consecutive 2 h intravenous infusions of 2,000 mg HRS-8427, the *C*_max_ in AM was 1.29 µg/mL, and the AUC_0–8_ was 6.11 h·μg/mL. In comparison, after a single 1 h intravenous infusion of cefiderocol, the *C*_max_ in AM was 1.23 µg/mL, and the AUC_0–6_ was 5.203 h·μg/mL (26). This suggests that the PK of HRS-8427 in AM are comparable to those of cefiderocol.

This study demonstrates that HRS-8427 exhibits favorable safety and tolerability profiles in healthy subjects. The overall incidence of TEAEs was 78.3% (29/37), which is comparable to clinical trial data for cefiderocol in healthy subjects (70%, 14/20) (26). Notably, 89.7% (26/29) of the TEAEs were mild in severity, with only three events classified as moderate, one of which (lymphadenitis) was deemed unrelated to the study drug. Among the 29 subjects who experienced at least one TEAE, the incidence was similar to that observed with cefiderocol in healthy subjects. PK monitoring revealed no clinically significant changes in serum creatinine levels in either the single or multiple-dose groups, indicating superior renal safety for HRS-8427.

Previous studies have demonstrated that cefiderocol exhibits favorable intrapulmonary PK in hospitalized patients with ventilator-associated pneumonia, achieving concentrations in the ELF sufficient to treat Gram-negative bacteria (MIC ≤ 4 mg/L) (29). *In vitro* studies of cefiderocol show potent activity against various resistant strains, including extended-spectrum β-lactamase-producing bacteria, carbapenem-resistant pathogens, and difficult-to-treat *Pseudomonas aeruginosa* and *Acinetobacter baumannii* (30). Clinical research has confirmed the safety and efficacy of cefiderocol in the treatment of carbapenem-resistant *Acinetobacter baumannii* infections (31). The 2024 guidelines from the Infectious Diseases Society of America (IDSA) recommended cefiderocol as a first-line or alternative treatment for carbapenem-resistant bacterial infections (32). As a novel siderophore cephalosporin, HRS-8427 demonstrates significant PK advantages, with a *T*_1/2_ approximately 1.8 times longer than that of cefiderocol and an *in vivo* exposure approximately 4.8–5.6 times greater. These enhanced PK properties may allow for reduce dosing frequency, providing a new option for optimizing the treatment of resistant Gram-negative bacterial infections.

The limitations of this study include the fact that, although PK data for HRS-8427 in plasma and its intrapulmonary penetration were obtained in healthy subjects, significant physiological and pathological differences exist between healthy subjects and patients with pneumonia, which may affect the drug’s alveolar penetration efficiency. Therefore, further clinical trials are warranted to confirm the intrapulmonary penetration of HRS-8427 in patients with pneumonia. Additionally, the BAL sampling density was relatively low, with only four sampling points in the single-dose group and three in the multiple-dose group. This limitation impacts the precision of the concentration-time profiles in the lungs, highlighting the need for further development of a pulmonary PK model for HRS-8427 to more accurately characterize its PK in pulmonary tissue.

Although previous *in vitro* studies determined the plasma protein binding rate of HRS-8427 to be 55.6%, with a corresponding unbound fraction of 44.4% (unpublished data), the present study did not directly measure the unbound drug concentration in plasma *in vivo*. Therefore, while the *in vitro* data provide an estimated basis for interpreting the ELF-to-plasma penetration ratio, the actual unbound drug concentration *in vivo* may differ from the *in vitro* findings. Notably, the ELF concentration range of HRS-8427 observed in the multiple-dose group was sufficient to cover the MIC₉₀ values of major pathogens, including *Escherichia coli*, *Klebsiella pneumoniae*, *Pseudomonas aeruginosa*, and *Acinetobacter baumannii*. However, the absence of measured unbound concentrations in both plasma and ELF limits the PK/PD evaluation of HRS-8427 in this study. Future studies should consider directly measuring the unbound plasma concentrations to enable a more accurate assessment of the PK/PD relationship of HRS-8427.

Notably, multiple-dose of HRS-8427 resulted in greater pulmonary drug accumulation, as reflected by elevated ELF concentrations and higher ELF/plasma ratios compared with single-dose administration. This pattern suggests a potential time-dependent increase in intrapulmonary penetration, which could enhance efficacy in pulmonary infections. Nevertheless, the overall extent of pulmonary exposure remained lower than that reported for several antibiotics commonly used to treat pneumonia, and accumulation within alveolar macrophages was limited. These findings indicate that the applicability of HRS-8427 for pulmonary indications remains to be further substantiated by future clinical trial results. However, its favorable systemic exposure profile and ongoing clinical evaluation in complicated urinary tract infections indicate that it may hold promise for alternative therapeutic applications.

In summary, intravenous administration of 1,500 mg HRS-8427 over 2 h as a single dose and 2,000 mg in multiple doses was well tolerated and demonstrated a favorable safety profile in healthy Chinese adults. Following multiple-dose administration, HRS-8427 exhibited increased ELF concentrations and pulmonary permeability, suggesting potential for improved lung exposure over time. A 2,000 mg dose of HRS-8427 achieved concentrations sufficient to exceed the MIC_90_ values of key Gram-negative pathogens, including *Klebsiella pneumoniae*, *Pseudomonas aeruginosa*, and *Acinetobacter baumannii*, and may represent an effective regimen for the treatment of pulmonary infection-related indications. The findings of this study support further clinical trials of HRS-8427 in patients with Gram-negative bacterial infections, including those beyond the pulmonary compartment such as complicated urinary tract infections.
